# Improving the Well-being of Adolescents With Type 1 Diabetes During the COVID-19 Pandemic: Qualitative Study Exploring Acceptability and Clinical Usability of a Self-compassion Chatbot

**DOI:** 10.2196/40641

**Published:** 2023-05-05

**Authors:** Anna Boggiss, Nathan Consedine, Sarah Hopkins, Connor Silvester, Craig Jefferies, Paul Hofman, Anna Serlachius

**Affiliations:** 1 Department of Psychological Medicine Faculty of Medical and Health Sciences University of Auckland Auckland New Zealand; 2 Department of Psychology and Neuroscience, Auckland University of Technology Auckland New Zealand; 3 Starship Children's Health Auckland City Hospital Auckland New Zealand; 4 Liggins Institute University of Auckland Auckland New Zealand

**Keywords:** self-compassion, chatbot, conversational agent, artificial intelligence, adolescence, type 1 diabetes, mental health, digital health, psychosocial interventions, COVID-19, mobile phone

## Abstract

**Background:**

Before the COVID-19 pandemic, adolescents with type 1 diabetes (T1D) had already experienced far greater rates of psychological distress than their peers. With the pandemic further challenging mental health and increasing the barriers to maintaining optimal diabetes self-management, it is vital that this population has access to remotely deliverable, evidence-based interventions to improve psychological and diabetes outcomes. Chatbots, defined as digital conversational agents, offer these unique advantages, as well as the ability to engage in empathetic and personalized conversations 24-7. Building on previous work developing a self-compassion program for adolescents with T1D, a self-compassion chatbot (COMPASS) was developed for adolescents with T1D to address these concerns. However, the acceptability and potential clinical usability of a chatbot to deliver self-compassion coping tools to adolescents with T1D remained unknown.

**Objective:**

This qualitative study was designed to evaluate the acceptability and potential clinical utility of COMPASS among adolescents aged 12 to 16 years with T1D and diabetes health care professionals.

**Methods:**

Potential adolescent participants were recruited from previous participant lists, and on the web and in-clinic study flyers, whereas health care professionals were recruited via clinic emails and from diabetes research special interest groups. Qualitative Zoom (Zoom Video Communications, Inc) interviews exploring views on COMPASS were conducted with 19 adolescents (in 4 focus groups) and 11 diabetes health care professionals (in 2 focus groups and 6 individual interviews) from March 2022 to April 2022. Transcripts were analyzed using directed content analysis to examine the features and content of greatest importance to both groups.

**Results:**

Adolescents were broadly representative of the youth population living with T1D in Aotearoa (11/19, 58% female; 13/19, 68% Aotearoa New Zealand European; and 2/19, 11% Māori). Health care professionals represented a range of disciplines, including diabetes nurse specialists (3/11, 27%), health psychologists (3/11, 27%), dieticians (3/11, 27%), and endocrinologists (2/11, 18%). The findings offer insight into what adolescents with T1D and their health care professionals see as the shared advantages of COMPASS and desired future additions, such as personalization (mentioned by all 19 adolescents), self-management support (mentioned by 13/19, 68% of adolescents), clinical utility (mentioned by all 11 health care professionals), and breadth and flexibility of tools (mentioned by 10/11, 91% of health care professionals).

**Conclusions:**

Early data suggest that COMPASS is acceptable, is relevant to common difficulties, and has clinical utility during the COVID-19 pandemic. However, shared desired features among both groups, including problem-solving and integration with diabetes technology to support self-management; creating a safe peer-to-peer sense of community; and broadening the representation of cultures, lived experience stories, and diabetes challenges, could further improve the potential of the chatbot. On the basis of these findings, COMPASS is currently being improved to be tested in a feasibility study.

## Introduction

### Background

Before the COVID-19 pandemic, adolescents with type 1 diabetes (T1D) had already experienced high rates of psychological distress and self-management challenges [1]. T1D is an autoimmune disorder that requires lifelong insulin therapy. Maintaining optimal self-management of T1D involves adherence to a complicated routine of daily self-administration of insulin and monitoring diet, energy expenditure, and blood glucose levels, with an estimated average of 180 daily self-management decisions [2]. In addition, adolescents must learn how to make these complex routines flexible enough to integrate into their daily lives, including school, hobbies, and other activities [3], while concurrently navigating developmental changes and demands. As a result, diabetes self-management tends to deteriorate during adolescence [4], and the prevalence of psychological disorders among adolescents with T1D is estimated to be 2 to 4 times greater than that of their general peers [1]. In Aotearoa, New Zealand, recent studies have estimated the rates of diabetes distress and disordered eating behaviors to be as high as 24% and 31%, respectively [5], with rates of psychological distress and self-management difficulties disproportionately affecting Indigenous Māori youth and those with socioeconomic deprivation [5-7].

Understandably, the COVID-19 pandemic has added further barriers to maintaining optimal glycemic targets and challenges to mental health in this population. As T1D is a condition that depends on multiple daily tasks, the COVID-19 pandemic has caused extensive disruption to daily routines and standard care. Commonly reported disruptions include restricted access to face-to-face health care; periods of home isolation; reduced physical activity owing to confinement; fear of more considerable COVID-19–related health risks; lack of social support from family, friends, and their diabetes care teams; and changes in eating behaviors and routines [8,9]. Although research has not specifically quantified the impacts of the COVID-19 pandemic on adolescents with T1D, the effects of the pandemic among people with T1D are becoming increasingly clear. For example, a high prevalence of eating and sleep disorders has been reported in people with diabetes [10,11]. In addition, although the reported impacts of the pandemic on diabetes management vary by country and insulin treatment technology, multiple countries have reported an increase in diabetic ketoacidosis frequency [12,13] and suboptimal glycemic variability [14,15]. Although a number of novel digital well-being initiatives became available during the COVID-19 pandemic [16-19], none addressed the unique challenges that adolescents with T1D face.

However, although the COVID-19 pandemic has compounded this problem, standard diabetes care was already struggling to provide adequate psychosocial support in this population [20,21]. Furthermore, despite the existence of evidence-based psychosocial interventions, they are rarely integrated within standard care because of ongoing funding constraints and clinician availability [20,22]. With the COVID-19 pandemic disrupting access to face-to-face health services and placing additional demands on systems, the existing constraints and lack of therapist availability are compounded. With worsening mental and physical health outcomes, digital interventions potentially offer this population a more accessible and clinically usable approach. However, a recent systematic review of digital interventions for youth with diabetes revealed an urgent need for evidence-based digital interventions to target psychological well-being [23]. To address the need for clinically usable psychological interventions for this sample and ongoing challenges related to COVID-19, we have recently developed a digital intervention based on self-compassion for adolescents with T1D.

### Self-compassion Chatbot

Self-compassion, which focuses on being kind and understanding toward our failures and hardships instead of being harsh and self-critical [24], is a promising treatment approach that appears to be highly relevant to the self-criticism that often accompanies attempts to adhere to complex self-management regimens, such as those in T1D. Face-to-face self-compassion interventions have demonstrated improvements in psychological health in adults [25] and adolescents [26] as well as in glycemic stability in adults with diabetes [27]. Our previous research found self-compassion to be an acceptable approach to improving mental and physical health in adolescents with T1D; however, face-to-face delivery demonstrated serious feasibility issues [28]. Our follow-up qualitative research, exploring perceptions regarding a possible digital adaption, demonstrated that a chatbot adaption was the preferred digital platform among adolescents with T1D [29].

A chatbot is defined as an automated computer program designed to simulate and process natural human conversation, allowing humans to interact with content as if they were communicating with a real person [30]. As a delivery modality, chatbots offer unique advantages over face-to-face therapy and other digital tools, including 24-hour availability, accessibility, remote delivery, scalability, and the capability to respond with personalized and empathetic responses in real time when they are needed. The evidence for their utility in supplementing standard care is growing. A recent review of mental health chatbots found improvements in symptoms of depression, anxiety, and general coping skills [30]. Chatbots have also been used for youth with health conditions, with a positive psychology skills chatbot showing reductions in anxiety for young adult cancer survivors [31]. In addition, chatbots have also shown acceptability and efficacy for improving self-management in a variety of health care settings, such as pediatric asthma self-management [32], chronic pain [33], and irritable bowel disease [34]. Although the efficacy of chatbots for youth with T1D remains unknown, the existing literature demonstrates that chatbots offer unique modality advantages and the potential to be feasibly embedded into existing diabetes technology to minimize patient burden.

Considering the ongoing impact of the COVID-19 pandemic on the preexisting high prevalence of psychological distress and self-management difficulties in this population and the unique utility of chatbots as a treatment modality, our research team developed a chatbot app intervention (called “COMPASS”) for adolescents aged 12 to 16 years with T1D in Aotearoa, New Zealand. The COMPASS chatbot is designed to deliver daily content in 14 conversational lessons daily across 2 weeks, aimed at facilitating self-compassion coping skills for adolescents with T1D. Accordingly, we first conducted focus groups to examine the user acceptability and potential clinical utility of the COMPASS chatbot app. As health care professionals commonly introduce and recommend mobile health apps to their patients [35], their perspective on a potential intervention for their patients was examined alongside the views of adolescents with T1D themselves.

## Methods

### Study Design

This qualitative study used focus groups and one-on-one interviews to examine the acceptability and the potential clinical utility of the COMPASS chatbot app in adolescents (aged 12-16 years) with T1D and their diabetes health care professionals. Focus groups were chosen for adolescents to encourage interactive, free-flowing conversations, whereas the choice of focus groups or one-on-one interview formats were offered to health care professionals to accommodate schedules during the COVID-19 Omicron surge in Aotearoa, New Zealand, with most health care professionals experiencing redeployment and increased clinical demands. As the prototype is still in the development phase and our focus is on the acceptability and the potential clinical utility of the included content and features, participants were shown screen recordings of the chatbot and were not given access to test it on their own devices.

### Ethics Approval

The study received ethics approval from the Health and Disability Ethics Committee, New Zealand (Ref: A+9284), and all participants provided informed consent or assent. Recruitment started on February 23, 2022, and was completed on April 4, 2022. Focus groups and interviews were conducted between March 4, 2022, and April 4, 2022. The methods were reported following the COREQ (Consolidated Criteria for Reporting Qualitative Studies) [36].

### Self-compassion Chatbot

#### Overview

The COMPASS chatbot delivers content designed to facilitate self-compassion coping skills for adolescents with T1D in 14 conversational lessons provided daily for 2 weeks. The preliminary conversational content and decision tree for COMPASS were developed by the first author (AB), with feedback from the coauthor SH and the University of Auckland’s Health Advances Through Behavior Intervention Technologies team [37].

#### Content

**Figure 1:**
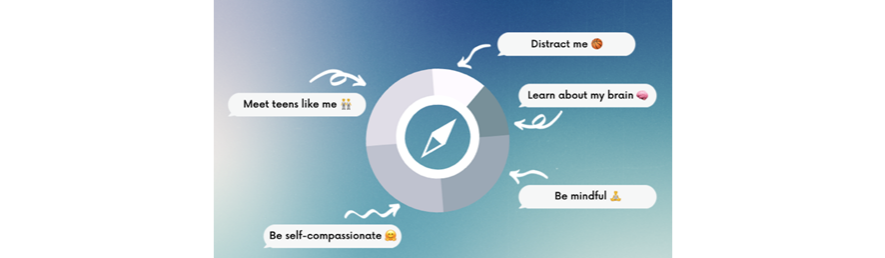
An overview of the types of activities included in the self-compassion chatbot.

The lesson content for the COMPASS chatbot was adapted from the evidence-based standardized 8-week adolescent self-compassion program [13] and our research teams’ brief 2-week adaption of the 8-week program for the specific challenges experienced by adolescents with T1D [28,38]. Most lessons are structured around the three components of self-compassion: (1) mindfulness (defined as having a balanced awareness of thoughts and feelings and grouped together in the chatbot as the “be mindful” activities), (2) common humanity (defined as acknowledging that challenges and imperfectness are part of being human and grouped together in the chatbot as the “meet teens like” activities), and (3) self-kindness (defined as being caring and understanding toward oneself and grouped together in the chatbot as “be self-compassionate”) [24]. Figure 1 shows an overview of the chatbot’s content. The chatbot also contains a psychoeducational activity (called “learn about my brain”) and distraction activities, which include rugby, netball, soccer, and basketball swipe sports games. Table 1 presents an outline of each activity included and a brief description.

**Table 1:** The 14 daily activities currently included in the first self-compassion chatbot prototype, presented in the order they are suggested to the user under each subheading of activities.

Activity category			Activity name and a brief description
**Compulsory introductory activities**			
	Be self-compassionate	“Self-compassion 101,” an introductory activity using the “how I treat a friend versus how I treat a friend” exercise to explain the concept of the inner critic and what self-compassion is “The three steps to self-compassion,” an introduction activity practicing using the 3 components of self-compassion for a current stressor (mindfulness, common humanity, and self-kindness)	
**Psychoeducation**			
	Learn about my brain	“Learn about my brain,” a psychoeducation activity explaining adolescent brain development and emotion regulation systems [39] to establish why self-compassion can help with managing difficult emotions	
**Mindfulness**			
	Be mindful	“Grounding exercise,” a mindfulness activity focusing on paying attention to our different senses “Check-in meditation,” a mindfulness activity focusing on checking in on our emotions and giving self-compassion toward those feelings “Music meditation,” a mindfulness activity focusing attention on the different instruments, tones, and sounds in music “Compassionate body scan,” a mindfulness activity focusing attention on the different areas of our bodies and giving gratitude to what our body does for us instead of what it looks like	
**Common humanity**			
	Meet teens like me	“Meet teens like me,” an activity using videos from older teenagers, young adults, and famous New Zealand figures with T1D talking about common struggles to create a sense of common humanity “Using self-compassion for diabetes struggles,” an activity using the 3 steps of self-compassion for diabetes burnout	
**Self-compassion**			
	Be self-compassionate	“Motivating ourselves with self-compassion,” an activity on setting goals with self-compassion instead of self-criticism “Self-compassion myth busters,” an activity outlining common struggles to being self-compassionate and metaphors and activities to help overcome common misconceptions or challenges “Compassionate friend meditation,” an activity aimed at giving ourselves the same compassion and understanding a close friend or pet does “Re-writing inner critic statements,” an activity practicing using self-compassionate statements instead of critical ones “Comforting gestures,” an activity aimed at finding a gesture (ie, putting your hands over your heart) that feels comforting (also called “soothing touch”)	

#### Format and Functionality

The chatbot delivers prewritten conversational lessons mostly via decision tree “rule-based” programming [40]. Therefore, the chatbot content is predominantly in dialog format that is based on predetermined “quick options” (such as “yes,” “maybe,” “no,” “let’s try it,” “tell me more”) that branch out the conversation along the user-chosen path. However, the COMPASS chatbot uses some instances of artificial intelligence to identify emotions; whether diabetes management is suboptimal or optimal; and risk words to deliver personalized, empathetic, and relevant responses and adequate information to connect with further crises and mental health support services. Further format and functionality features of the current prototype version were informed by suggestions from a previous qualitative study conducted by our research team, which explored the functionality and content that adolescents with T1D wanted to see in future digital mental health programs [29].

**Figure 2:**
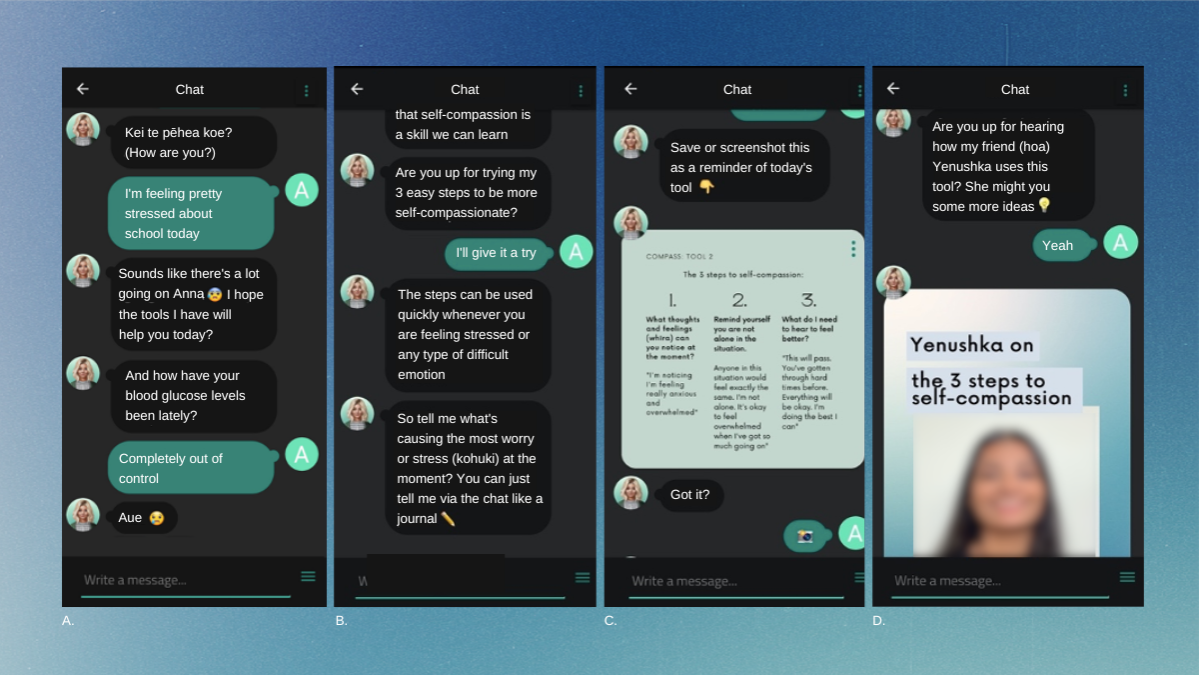
Example of the daily structure of the self-compassion chatbot, in sequential order of features and activities. A. Example of the emotion and diabetes check-in. B. Example of the introduction to a daily activity. C. Example of a summary infographic included in all daily activities. D. Example of lived experience videos included in some activities.

When the user first engages with the COMPASS chatbot, an onboarding module explains how the chatbot is structured and emphasizes that it is an automated person (not a real-life human). These early exchanges encourage participants to try all the different activities over the following 2 weeks, ask the user for a nickname to call them, and ask the user to set a time for the chatbot to send a daily notification to check in with the chatbot and complete an activity. The chatbot also allows the user to pick an avatar to talk to, with options representing a range of gender identities and ethnicities. Subsequently, the chatbot then begins an introductory activity before asking for feedback. After the first day, the chatbot follows a general structure of daily notification, emotion and diabetes check-in, choice of daily activity, and activity feedback (Figure 2 shows examples of each component). Although the chatbot can deliver a new activity every day, there is an option to repeat activities that the user has previously completed by either looking at a brief summary or performing the activity again. Each daily interaction with the chatbot was designed to last approximately 5 minutes.

The written content is enhanced by additional features and media. For example, each module contains a summary infographic at the end of each activity (seen in Figure 2) so that adolescents can save images of the tools that they found helpful. The activities are also enhanced by using audio-guided meditations, Graphics Interchange Formats (GIFs), and videos from older Aotearoa New Zealand teenagers, young adults, and celebrities with T1D talking about common struggles. Videos discuss topics such as diabetes burnout, feeling isolated from peers, navigating relationships with peers and diabetes inner critics, how to be self-compassionate when experiencing diabetes burnout, communicating with others who do not understand T1D, and how to manage T1D in sports contexts. To improve accessibility and usability, all videos contained subtitles, and all meditations had written message adaptions.

### Study Participants and Recruitment

A target sample size of approximately 15 to 20 adolescents with T1D (aged between 12 and 16 years) and 10 to 15 diabetes health care professionals were chosen as an achievable recruitment target. Informed by earlier qualitative work in this area [29] and reviews of qualitative research [41], we estimated that this sample size would allow data saturation to occur. However, if data saturation was not achieved, an ethical amendment would have been applied to expand the number of participants. The eligibility criteria for adolescents included the following: being aged between 12 and 16 years; living in Aotearoa, New Zealand; having a diagnosis of T1D for >6 months; having no diagnoses of serious developmental or psychiatric disorders; and being able to provide consent in English. In addition, we aimed to recruit at least 5 Māori and Pacific young people to allow for one group of solely Māori and Pacific participants. Diabetes health care professionals were eligible if they lived and worked in Aotearoa, New Zealand, and worked within a pediatric or young adult diabetes care team.

Adolescents who had participated in previous research [28,29] and had consented to be conducted for future research projects were invited to participate via email on a first-in-first-serve basis. Concurrently, study flyers were given to eligible participants in pediatric diabetes clinics in the Auckland region by endocrinologists, PH and CJ, who were involved in the study, and study flyers were posted in web-based communities, such as Diabetes Youth Auckland and Type 1 Diabetes Youth in Aotearoa New Zealand Facebook pages. Diabetes health care professionals were recruited by emailing diabetes clinics in Auckland and using diabetes research special interest groups throughout Aotearoa, New Zealand.

Participants who expressed interest in the study via email or the link on our study flyer were directed to a secure website, REDCap (Research Electronic Data Capture; Vanderbilt University) [42], to assess eligibility and provide informed consent or assent. At the time of consent or assent, adolescents were asked to provide demographic variables, such as age, gender, ethnicity, length of diabetes diagnosis, current insulin regimen, and whether or not they use a continuous glucose monitoring system. Similarly, diabetes health care professionals were asked for their gender, ethnicity, role within the diabetes team, and length of time for which they worked with youth with T1D. All participants were mailed a voucher for NZD $50 (approximately US $34.86) after completing their focus groups or interviews.

### Focus Group Procedures

Adolescent focus groups were conducted on the web using Zoom (Zoom Video Communications, Inc), with 3 to 5 adolescents per focus group and lasted up to 90 minutes. A total of 4 focus groups were conducted, with 1 focus group reserved solely for Māori and Pacific youth to ensure that their perspective on the cultural responsiveness of the app was emphasized. All adolescent focus groups were facilitated by the first author (AB, an Aotearoa New Zealand European female health psychology PhD candidate and a health psychology preintern) and another study author (CS, an Aotearoa New Zealand European or Māori male registered as a psychologist). Both focus group facilitators had experience in facilitating group sessions. Of note, ALB had existing relationships with some of the adolescent participants (11/19, 58%) who had participated in the previous research [28,29]. The adolescents were informed that ALB was completing the study for her PhD, which involved developing a digital well-being intervention for youth with T1D.

Diabetes health care professional interviews were conducted on the web using Zoom, depending on clinician availability, and lasted up to 90 minutes. All diabetes health care professional interviews were facilitated solely by AB. As mentioned earlier, ALB did have existing relationships with many diabetes health care professionals (9/11, 82%) because they participated in similar diabetes-related research and via professional clinical networks.

The focus groups and interviews followed a semistructured interview schedule devised by ALB and supervisors NSC and ASS. Participants were shown images and screen recordings from the chatbot prototype app and asked questions related to the domains included in the end user version of the mobile application rating scale, including engagement (eg, “How could we make this exercise more engaging?”), functionality (eg, “What features would you like to see added to the chatbot?”), aesthetics (eg, “What module do you like the look of the least?”), and information (eg, “Are there any topics you would like us to add?”) [43]. Questions were also included to gain feedback on cultural appropriateness (eg, “Do you think COMPASS is culturally appropriate for all young people with type 1 diabetes of different ethnic groups in New Zealand?” and “How do you think we could improve the cultural acceptability of the app?”). Furthermore, a summary of the key points and the possibility of data saturation was discussed between CS and ALB after each adolescent focus group. For diabetes health care professionals, this was conducted by ALB under the supervision of NSC and ASS.

### Qualitative Analysis

All interviews and focus groups were digitally audio recorded and transcribed. The transcripts were then analyzed using directed content analysis [44], a qualitative data analysis approach suited for focus groups and interviews with predetermined categories and research questions exploring an existing theory or framework. A framework of usability categories from the user version of the mobile application rating scale questionnaire was also used to assist in categorizing themes under those features and content that were disliked, liked, and desired for future addition. Similar to the approach used in a previous qualitative work [29], the analysis was based on the following predetermined research questions: “what do adolescents with T1D and their health care professionals like about COMPASS?” “what do adolescents with T1D and their health care professionals dislike about COMPASS?” and “what future additions do adolescents with T1D and their health care professionals desire in a second prototype of COMPASS?”

Using this approach, transcripts were first coded independently by ALB and CS using NVivo (QSR International), a qualitative data analysis computer software package. The coders built lists of liked, disliked, and desired content and features and started to group these across the different research questions by common themes. This was chosen because similar themes were observed across the 3 research questions. For example, adolescents reported to dislike memes or jokes that were not age appropriate, liked the discussion of relevant and age-appropriate stressors, and expressed a desire for age-appropriate humor and relevant topics to be discussed, forming the overall “relevant and age-appropriate” theme. The coders then agreed on the final themes and subthemes before conducting coding independently for the second round of analysis. Any discrepancies in the coding were resolved by consensus.

## Results

### Participant Demographics

A total of 19 adolescents consented or assented to participate, and no participants dropped out of the study. In total, 4 focus groups were conducted, ranging from 82 to 107 minutes in duration (mean 91.89, SD 10.67 minutes). Each group averaged 4 participants (mean 4.75, SD 0.96). Just over half of the participants used a pump for their insulin treatment (10/19, 53%) compared with injections (9/19, 47%), and most participants (14/19, 74%) used continuous glucose monitors. The number of years diagnosed with T1D ranged from 2.5 to 14.5 years (mean 7.63, SD 4.00 years). Ethnicity was divided between Aotearoa New Zealand European (13/19, 68%), Māori (2/19, 11%), Samoan (3/19, 16%), and Indian (10/19, 5%), and 58% (11/19) were female, a distribution that is broadly representative of the population living with T1D in Aotearoa, New Zealand: 51% female and 75% Aotearoa New Zealand European [45].

A total of 11 diabetes health care professionals consented to participate, and no participants dropped out of the study. In total, 2 focus groups, 1 with 18% (2/11) of participants and 1 with 27% (3/11) of participants, and 9 one-on-one interviews were conducted, ranging from 70.24 to 103.48 (mean 83.28, SD 11.19) minutes in duration. Most participants were female (8/11, 73%) and identified as Aotearoa New Zealand European (9/11, 82%), followed by Samoan (1/11, 9%) and Irish European (1/11, 9%). Health care professionals were from various backgrounds, including diabetes nurse specialists (3/11, 27%), health psychologists (3/11, 27%), dieticians (3/11, 27%), and endocrinologists (2/11, 18%), with an average of 13.97 (SD 11.58) years of experience working in diabetes management.

### Qualitative Findings

#### Adolescents

##### Overview

Generally, adolescents rated COMPASS positively, with 68% (13/19) saying they would recommend COMPASS to a friend with T1D. The remaining 32% (6/19) of participants voted yes if the suggested improvements highlighted in the themes below, personalization, self-management support, relevant and age-appropriate content, ease of use, and connectivity with others, were included (Table 2). Overall, when asked what their favorite module was, adolescents most commonly reported “meet teens like me” (chosen by 8/19, 42%), “distract me” (3/19, 16%), and “be self-compassionate” (3/19, 16%). Conversely, the least appealing modules were reported to be “learn about my brain” (8/19, 42%) and “be mindful” (7/19, 37%).

**Table 2:** Summary of key qualitative themes, with example quotations and their corresponding prevalence.

Participant group and themes^a^			Prevalence—Participants, n (%; comments, content coverage)		Example quotation
**Adolescents (N=19)**					
	1. Personalization	19 (100; in 51 comments, 29.62% content coverage)		“Just really add more ability to personalize it for what you wanna talk about and what you wanna do. Even with things like notifications or even the colors, or if you want it to link with your diabetes team or not” (male aged 15 years).	
	2. Self-management support	13 (68; in 39 comments, 30.89% content coverage)		“If we could have something like interactive with a video. It could start off, oh you’re low and then it has a series of options you could choose of different things to try out... I think it’s a good idea to give tips to help you manage it yourself” (female aged 12 years).	
	3. Relevant and age appropriate	14 (74; in 26 comments, 17.03% content coverage)		“If you have stuff like gifs or like memes I think it’s sort of risky I guess. Like you have to kind of, you gotta figure out what most people would enjoy. Like not, 'cause you can see some memes and then they’re like really old, like talking about weird things you haven’t even heard of. So I think it’s sort of gotta be like more, yeah, topical and current I guess” (male aged 13 years).	
	4. Ease of use	14 (74; in 26 comments, 10.94% content coverage)		“I think that with the videos, like the keyword type of thing would be helpful. Like having a search bar for either the information or the videos or whatever. Just because also if you get a lot of videos you don’t wanna be scrolling trying to find the one that you were looking for, or the right thing for you” (female aged 16 years).	
	5. Connectivity with others	14 (74; in 24 comments, 11.52% content coverage)		“I think like having a moderated discussion board with other members would be a good idea, just to like see how everyone’s doing and if anyone’s struggling. Just knowing that there’s others like me, we’re here too for them if there’s anything they need, advice or just, yeah. We’re all in the same shoes I guess” (male aged 16 years).	
**Diabetes health care professionals (N=11)**					
	1. Clinical utility	11 (100; in 52 comments, 36.29% content coverage)		“I think it could help us by giving them a walk-through of what to do, say they say their blood glucose is way out of control, it could automatically go down the route first of basic things like have you had your insulin today, have you checked ketones, just some simple safety things and brief suggestions” (diabetes nurse specialist).	
	2. Breadth and flexibility of tools	10 (91; in 48 comments, 35.26% content coverage)		“The self-compassion content here is awesome and would be relevant to almost anyone I see but I would probably like to see more about problem-solving and what are your values and how do we get you to live towards those and your diabetes management comes under that” (health psychologist).	
	3. Cultural appropriateness	8 (73; in 19 comments, 13.88%)		“I think just having an even broader spectrum of cultures and stories from families with different backgrounds. So that then it’s relatable for everyone” (diabetes nurse specialist).	

^a^Key themes are organized by how many participants reported the theme in each participant group.

##### Personalization

All participants (19/19, 100%) emphasized personalization of both content and features as being important to them, the most prevalent theme for the adolescent sample. Although COMPASS was able to personalize notification timings, responses, and content, adolescents mentioned wanting to be able to personalize a wider range of content and features; for example, being able to personalize the included games, apps, background color schemes, and hobbies referenced in examples and videos; design their own chatbot avatar to talk to; and personalize the number of notifications further (eg, the amount per day and week); and change settings when their routine changes (eg, if they become sick or go on holidays).

##### Self-management Support

Of 19 adolescent participants, 13 (68%) expressed the desire for the chatbot to assist them with independent self-management, a feature not offered in the current prototype. Participants commonly said it was difficult to find reliable and trusted sources of information to support new scenarios, such as how to manage T1D when the test result is positive for COVID-19 or considerations when starting a new hobby (such as weightlifting). For example, “otherwise I’m on 20 different websites that are telling me different scary things and that is not easy and really stressful” (female aged 16 years). A problem-solving feature was also commonly suggested, with the chatbot offering things one could try to get in their desired range in a compassionate tone rather than asking “how have your blood glucose levels been lately?” Participants also emphasized the added benefit if the app could also integrate with their other diabetes technologies to help with problem-solving. For example:

[I]t would be so good just having one app with everything there and just being able to look at your levels and have something there to give you ideas on how you could get your levels down.

Female aged 12 years

In the focus group with Samoan and Māori adolescents, including more features to assist with self-management, such as reminders to take their diabetes kits to school and the option to enter blood glucose levels into the app, were identified as being especially important.

##### Relevant and Age-Appropriate Content

Another central theme reported by most participants (14/19, 74%) involved not only ensuring that the content of the chatbot remains relevant and age appropriate, especially regarding humor, GIFs, and slang, but also including relevant topics. For example, drinking and drugs were highlighted as topics that were appropriate for this platform, where more private topics could be discussed safely. For example:

[I]f it could include like drugs and alcohol and diabetes cause we don’t get that information given to us unless we ask our diabetes team and that’s kinda awkward...like some people with diabetes don’t even know that alcohol can affect them differently right...so getting that information might help them make better decisions as to whether they wanna drink or take drugs.

Female aged 16 years

##### Ease of Use

Similarly, 74% (14/19) of the adolescent participants suggested additional features to increase usability and autonomy, such as collating all the videos in one place, providing a search bar function if they wanted to find resources or videos on a specific topic, having a tab for offline resources, and being able to skip activities or videos more easily if they wanted to try something else. Some participants also mentioned that adding an element of artificial intelligence in which the algorithm would learn what videos and topics you like to watch and then tailor future topics and activities would make the chatbot easier to use and more engaging.

##### Connectivity With Others

Connecting with others with T1D was also identified as being important (mentioned by 14/19, 74% of participants). Although the videos of lived experiences in the chatbot were liked, most participants expressed a need for the social connection to go further and suggested having a moderated forum where people could post questions and share advice with others. For example:

I think having, like a forum like Reddit, so you know how Reddit has subchannels and stuff, and you can put a question and people can upvote and reply to it...just having the reassurance that there are other people like you who could help and talk to when you need to.

Female aged 15 years

##### Minor Themes

A total of 2 minor themes were also identified in the adolescent data: increased privacy (mentioned by 6/19, 32% of participants) and increased representation (mentioned by 3/19, 16% of participants). Although only 5% (1/19) of participants reported that they shared a phone with others in their household, privacy from friends or siblings using or looking through their phone when they were not looking was mentioned as being important. For example:

[J]ust in case friends or siblings or someone goes onto your phone maybe have like a login password or something for the whole app...that way people just can’t go in and like see the texts and your mindfulness and all that.

Male aged 15 years

Second, although all the adolescent participants said that they thought the app was culturally appropriate for all young people with T1D of different ethnicities in Aotearoa, New Zealand, 16% (3/19) of participants expressed a desire for more representation in the included content and videos in terms of ethnicities and other languages, such as Samoan

#### Health Care Professionals

Overall, all health care professionals (11/11, 100%) rated the app positively and stated that they would recommend it to their patients. In the following sections, the identified strengths and suggestions for improvements are highlighted in the overall themes.

##### Clinical Utility

All participants (11/11, 100%) referenced the theme of clinical utility, both in terms of the COMPASS chatbot being useful in complementing current standard care (notably within the context of ongoing COVID-19 pandemic) and also in identifying additional features and applications to bring more benefit. Integration with diabetes technology to assist with problem-solving was offered as a suggestion, along with comments relating to COMPASS’ potential utility to support parents or caregivers and older young adults. Clinical integration as a feature was discussed from various perspectives; one health care professional thought that clinical integration would be helpful, whereas others thought it was unfeasible and had negative experiences with integration attempts in the past. One dietitian suggested that a compromise could be the ability to ask the chatbot to remember any questions or topics that the adolescents wanted to ask their diabetes team and then set a reminder before their next clinic appointment.

##### Breadth and Flexibility of Tools

Although all health care professionals thought self-compassion was relevant, flexible, and clinically useful to their patients, some participants (10/11, 91%) expressed a desire for a greater range of tools to be included. These included adding more diabetes-specific content and examples (such as applying tools to more diabetes-specific stressors such as fear of highs or lows), a broader range of evidence-based tools (such as values and benefit finding), more tools for emotional distress (such as more guided breathing illustrations), and a broader range of lived experience videos (eg, being self-conscious about people noticing visible insulin pumps or continuous glucose monitors and experiences with alcohol and T1D self-management). Some participants also identified the chatbot app as a suitable place to put commonly used tools to make it “the go-to place for those with T1D in Aotearoa” (mentioned by a diabetes nurse specialist), such as etiquette cards (ie, how to communicate with people who do not understand the challenges of T1D); easy math exercises for glucose levels and correction doses; and relevant information, such as updated diabetes COVID specific or managing diabetes self-management under exam settings. Some participants also expressed the desire for the chatbot to be more flexible and autonomous, for example, if an adolescent wanted to skip a check-in 1 day or conversely be talked through difficult feelings in more depth before moving on to exercise.

##### Cultural Appropriateness

In addition, of the 11 participants, 8 (73%) mentioned the need to increase the representation of different ethnicities and stories in lived experience videos. Tailoring self-compassion content to match cultural values was also identified as an area for possible improvement to further address some of the misconceptions about self-compassion. For example:

[F]or some, it could be hard to understand that you can still be self-compassionate and uphold and respect your family and cultural values...it doesn’t mean that you’ve got to relinquish any of your personal responsibilities or ignore your personal health...looking after your own hauora [health and wellbeing in te reo Māori] is going to make you more responsive and able to care for your family.

Health psychologist

Although some participants identified the integration of te reo (one of New Zealand’s national languages) as a strength of the chatbot, health care professionals also wanted to see other languages included, such as Tongan and Samoan.

##### Minor Themes

In addition, 3 minor themes were identified: reassuring tone (mentioned by 7/11, 64% of participants in 10 comments), creating a safe sense of community (mentioned by 5/11, 45% of participants in 10 comments), and equity of access (mentioned by 4/11, 36% of participants in 6 comments). The reassuring and validating tone of COMPASS was identified as a strength, and the importance of keeping the tone similar for the future was also emphasized. For example:

[Y]ou wanna balance it with being like a helpful friend rather than being a nagging parent or diabetes nurse.

Diabetes nurse specialist

Creating a safe sense of community was mentioned as both a strength of the videos but also in a future moderated forum or chat to ease the isolation health care professionals commonly see in their patients. Finally, equity of access was also mentioned as an essential consideration, ensuring that the app had available offline resources and was free to download. The provision of phones was also identified as necessary for inclusion in the future funding.

## Discussion

### Principal Findings

Building on the need for clinically usable psychological interventions during the challenging period posed by the COVID-19 pandemic, this study was designed to develop and examine the acceptability of COMPASS for adolescents with T1D and their health care professionals. Overall, most participants rated COMPASS positively as being appealing, engaging to use, relevant, and complementing standard care. The qualitative results illustrated the areas of most importance to adolescents with T1D, including personalization, support with self-management, relevant and age-appropriate content, ease of use and connectivity with others and their health care professionals, such as clinical utility, breadth and flexibility of tools, and cultural appropriateness. Although the 2 groups demonstrated different overall themes, several suggestions and features were shared across them, such as including more features to support self-management (eg, integration with diabetes technology, problem-solving assistance, and a source of up-to-date information); increasing the range of tools and topics (beyond self-compassion); creating a safe peer-to-peer community; and broadening the representation of different cultures, lived experience stories, and diabetes challenges.

These highlighted themes and features are consistent with previously identified effective and desirable chatbot and digital intervention components, such as personalization, relevant and age-appropriate content, and peer-to-peer support features. Previous reviews of digital interventions have noted the importance of these features both in the effectiveness and engagement of youth [46]. In addition, in our previous qualitative study among adolescents with T1D, perspectives on different digital intervention modalities, personalization, and peer-to-peer support were also identified as desirable features [29]. However, personalization was a more prevalent theme in this study, with desires for personalization extending to the personalization of content based on hobbies and interests and the people and voices in the videos and meditations, perhaps indicative of the potential utility chatbots can offer. The emphasis on the importance of personalization in chatbot delivery also mirrors related studies examining the user acceptability of chatbots among youth samples [47] and mental health chatbots more broadly [48]. Connectivity with other peers also emerged as an important theme, which was referenced in the previous study [29]. Although most participants liked the lived experiences videos, they still expressed the need to give and receive peer-to-peer advice and did not perceive discussions being moderated as a potential barrier to engagement. Interventions not being age appropriate or “cringey” were also referenced in the previous study, further emphasizing the importance of co-designing interventional content and delivery with adolescents. The desire for peer support is commonly referenced across youth interventions, especially among those with chronic health conditions [49]. This pattern is consistent with the notion that peer support features should be included in chatbot interventions in this group.

Another common feature suggested by both samples was the inclusion of features to support diabetes decision-making and self-management. Recent studies have investigated the efficacy and acceptability of chatbots in supporting users to make clinical and health-related decisions and problem-solving in contexts such as cancer care [50], nursing students [51], support for older adults [52] and problem-solving for anxiety and depression [53,54]. More specifically, recent work in diabetes care has explored using a chatbot to support individuals aged 15 to 18 years with T1D in interpreting blood glucose levels, suggesting what to do in hypoglycemia events, and providing self-care behavior reminders [55]. The results of this study show that such support is desired by both adolescents and their health care professionals and may offer further benefits when combined with psychological support.

### Limitations

Although these identified strengths of COMPASS and desired future additions propose a novel and unique adapted chatbot, several possible sources of bias should be considered when interpreting our results. Many participants in both samples had previously been involved in focus groups or self-compassion interventions conducted by the first author (AB) or were known via professional networks. Although such connections help recruitment, they may also bias opinions and feedback to be more complimentary. Second, owing to the delays in app development and our focus on the acceptability and perceived potential clinical utility and usability of the chatbot features and content, participants were only shown screen recordings of the chatbot, which may have restricted the richness of the feedback we received. However, the subsequent planned study includes focus groups to explore the user experience in more detail. In addition, although data saturation was reached and the adolescent participants were broadly representative of the adolescent population with T1D in Aotearoa, New Zealand, the sample may not have been representative of the breadth of challenges those with T1D experience and those who experience additional barriers to accessing standard care. It is important for future studies to include provisions for supplying phones and features, such as being able to log and track blood glucose levels, and to reduce inequities between those who have technology, such as smartphones and continuous glucose monitors, and those who do not have or face barriers in accessing standard care. In addition, incorporating feedback at each stage of the development from a representative range of users to ensure culturally appropriate approaches are being used, especially for Indigenous populations [56].

### Novel Contributions

Despite these considerations, this study has several strengths and novel contributions. To our knowledge, the COMPASS chatbot is the first chatbot to deliver psychological tools to adolescents with T1D, offering a novel intervention for this sample as a key strength of the study. Furthermore, including both adolescents and their health care professionals provided a deeper understanding of the potential acceptability and potential clinical utility of chatbots for this sample. As health care professionals commonly introduce their patients to mobile health apps [35], examining their perspective on a potential intervention is useful in ensuring that they would be comfortable recommending a chatbot app to their patients. As highlighted earlier, the qualitative results also provided more support for previously identified desired features and additions of digital interventions for this population, such as personalization of content and peer-to-peer support features. In addition, the results also offer new insights into what adolescents and their health care professionals want in an adapted version of the COMPASS chatbot and also provide guidance for those developing digital interventions for youth with T1D. For example, including problem-solving capabilities and integration with diabetes technology to support self-management along with psychological support is a unique contribution to the literature.

### Conclusions

In conclusion, our study findings provide preliminary support for the acceptability and potential clinical utility of COMPASS for adolescents with T1D, and highlight important features to be included in future chatbot interventions for this group. The results highlight several shared features suggested by both adolescents and their health care professionals, such as problem-solving features and integration with diabetes technology to support self-management; increased personalization of content; the addition of moderated app user peer-to-peer support to ease isolation and increase connection to others; and increased representation of different cultures, lived experience stories, and diabetes challenges. As such, COMPASS is currently being updated ahead of being tested in a feasibility study. If shown to be feasible, the next step will be to test the COMPASS app for efficacy to determine whether it would assist in filling the gaps in both self-management and psychological support exacerbated by the COVID-19 pandemic in Aotearoa, New Zealand. Future applications could include extending this self-compassion–based tool to adolescents with T1D in the global population or more broadly to other chronic health conditions that present with frequent opportunities for self-criticism and for which supportive conversational assistance with problem-solving features could also reduce disease burden. Furthermore, if efficacy can be illustrated, the chatbot could be integrated with other technologies, such as wearables (eg, smart watches, continuous glucose monitors, or insulin pumps).

## Abbreviations

COMPASS: self-compassion chatbot
COREQ: Consolidated Criteria for Reporting Qualitative Studies
GIF: Graphics Interchange Format
REDCap: Research Electronic Data Capture
T1D: type 1 diabetes
